# What are the beliefs, attitudes and practices of front-line staff in long-term care (LTC) facilities related to osteoporosis awareness, management and fracture prevention?

**DOI:** 10.1186/1471-2318-10-73

**Published:** 2010-10-08

**Authors:** Arthur N Lau, George Ioannidis, Yelena Potts, Lora M Giangregorio, Mary-Lou Van der Horst, Jonathan D Adachi, Alexandra Papaioannou

**Affiliations:** 1 Division of Rheumatology and Department of Medicine, St. Joseph's Healthcare and McMaster University, Hamilton, Ontario, Canada; 2 Departments of Clinical Epidemiology and Biostatistics and Medicine, McMaster University, Hamilton, Ontario, Canada; 3 St Joseph's Healthcare, Hamilton, Ontario, Canada; 4 Department of kinesiology, Waterloo University, Waterloo, Ontario, Canada; 5 Division of Geriatrics and Department of Medicine, Hamilton Health Science and McMaster University, Hamilton, Ontario, CIHR -Eli Lilly Chair Osteoporosis and Fracture Prevention, Canada

## Abstract

**Background:**

Compared to the general elderly population, those institutionalized in LTC facilities have the highest prevalence of osteoporosis and subsequently have higher incidences of vertebral and hip fractures. The goal of this study is to determine how well nurses at LTC facilities are educated to properly administer bisphosphonates. A secondary question assessed was the nurse's and PSW's attitudes and beliefs regarding the role and benefits of vitamin D for LTC patients.

**Methods:**

Eight LTC facilities in Hamilton were surveyed, and all nurses were offered a survey. A total 57 registered nurses were surveyed. A 21 item questionnaire was developed to assess existing management practices and specific osteoporosis knowledge areas.

**Results:**

The questionnaire assessed the nurse's and personal support worker's (PSWs) education on how to properly administer bisphosphonates by having them select all applicable responses from a list of options. These options included administering the drug before, after or with meals, given with or separate from other medications, given with juice, given with or without water, given with the patient sitting up, or finally given with the patient supine. Only 52% of the nurses and 8.7% of PSWs administered the drug properly, where they selected the options: (given before meals, given with water, given separate from all other medications, and given in a sitting up position). If at least one incorrect option was selected, then it was scored as an inappropriate administration. Bisphosphonates were given before meals by 85% of nurses, given with water by 90%, given separately from other medication by 71%, and was administered in an upright position by 79%. Only 52% of the nurses and 8.7% of PSWs surveyed were administering the drug properly. Regarding the secondary question, of the 57 nurses surveyed, 68% strongly felt their patients should be prescribed vitamin D supplements. Of the 124 PSWs who completed the survey, 44.4% strongly felt their patients should be prescribed vitamin D supplementation.

**Conclusion:**

Bisphosphonates are quite effective in increasing the bone mineral density of LTC patients, and may reduce fracture rates, but it is only effective if properly administered. In our study, proper administration of bisphosphonate therapy was less than optimal. In summary, although the education of health providers has improved since the mid-1990's, this area still requires further attention and the subject of future quality assurance research.

## Background

Osteoporosis is a disease leading to progressive decreases in bone mineral density (BMD), decreased bone strength and increased risk of skeletal fractures [1]. Specifically, vertebral and hip fractures pose devastating consequences for the osteoporotic patients. Approximately 30% of women will have sustained at least one vertebral fracture by the age of 75 [2]. There are over 700,000 incident vertebral fractures related to osteoporosis each year in the United States [2]. The estimated number of hip fractures worldwide in the year 2050 is estimated to increase to 6.3 million [3]. Both vertebral and hip fractures are associated with an increase mortality rate [4,5]. Therapy for osteoporosis may reduce mortality rates based on recent studies [6,7], it is crucial to emphasize the impact a vertebral or hip fracture can have. Approximately 75% of patients who present with a clinical vertebral fracture will experience chronic pain [8]. Vertebral fractures also have a significant impact on quality of life and functional impairment on the affected patients [9] and results in increased acute care use, analgesic use and higher care needs [10].

An important population to consider is the patients living in long term care (LTC) facilities. Compared to the general elderly population, those institutionalized have the highest prevalence of osteoporosis. Rates have been estimated to be as high as 79% in males and 86% in females over the age of 85 [11], and this translates to a higher incidence of fragility fractures. The annual incidence of hip fractures in LTC patients is estimated to be 4% (with a range of 2.3% to 6% annually) [12,13]. This annual incidence is estimated to be 3 to 11 times higher than similar aged individuals who live in the community [14,15]. It is also estimated that a vertebral fracture is present in 50% of LTC residents [16]. Elderly LTC residents have a 40% risk of death within 1 year after sustaining a hip fracture [17].

Canadian wide clinical practice guidelines do not specifically address the LTC population. However the unique needs of this population have been addressed by a consensus guide developed in Quebec. Global recommendations included: vitamin D, calcium, a comprehensive exercise program and the initiation of pharmacological treatment for residents at high risk. Specific recommendations are provided for screening, laboratory tests for secondary causes of osteoporosis and starting treatment including non-pharmacologic interventions, pharmacological treatments, and nutritional supplementation [18]. Although osteoporosis pose such a burden on these patients and the healthcare system, there remains a significant care gap between what are accepted to be recommended treatments and actual practice [18].

Aside from the physicians at the LTC facilities, the front line staffs (including the nurses and PSWs) plays a key role in influencing osteoporosis care in these facilities, and thereby helping to prevent future fractures in this high risk population. If properly educated on the risk of their patients to incur a fracture, and the current recommendations for these high risk patients, the front line staff has the ability to improve care through ensuring the recommendations are implemented, through education of the patients and families regarding fracture prevention, advocacy for their patients to ensure proper therapies are delivered, among other possibilities.

Currently, there is limited research on the perspectives of LTC staff on attitudes, practices and beliefs regarding osteoporosis awareness, management and fracture prevention. Several studies have investigated the attitudes and practices of the LTC facility administrative staff and physicians but there is currently limited research regarding the perspectives of the front-line staff. It is important to assess the current practice and education of front-line staff, as efficacious therapies and preventative strategies are not effective unless they are implemented and properly administered. The goal of this study is to determine how well nurses and PSWs) at LTC facilities are educated to properly administer bisphosphonates. A secondary question assessed was the nurse's and PSW's attitudes and beliefs regarding the role and benefits of vitamin D for LTC patients.

## Methods

### Study Population

Eight LTC facilities in Hamilton (Ontario, Canada) were surveyed. All front line staff who worked day shifts at each of these facilities was offered a survey. Those we aimed to include into the study included directors of care, directors of nursing, administrators, nurse educators, charge nurses, nurse practitioners, registered nurses, registered nursing assistants, physiotherapists, personal support workers, and students who trained at these facilities. Non-front-line staff and those who worked primarily night shifts were excluded from the study. For the current study we examined responses for the nurses and personal support workers. Personal support workers were individuals employed by the LTC facilities who aided with the resident's activities of daily living, which included medication administration among other activities. None of LTC staff surveyed were exposed to any specific education initiatives regarding osteoporosis management or fracture prevention. Also, the LTC staff members surveyed worked at different facilities from the ones associated with the study investigators. This study was approved by the local research ethics board at Hamilton Health Sciences.

### Questionnaire Administration

The project coordinator contacted each LTC home to introduce the survey to the administrator and scheduled a date to administer the questionnaire. The project coordinator was present for the administration of the questionnaire. The questionnaire's main objectives were to assess attitudes towards the link between osteoporosis and falls; existing management practices and specific osteoporosis knowledge areas. Item generation resulted from three sources: literature review, input from key informants and the team's clinical experience. Questions were reviewed and revised by content experts in clinical content and methodology, and face validity was reviewed by content experts. A 7 point Likert Scale was used to scale responses. It allowed for the collection of continuous data, which provides a gain in reliability compared to dichotomous responses [19]. The 7 point scale maximizes the amount of information, which most individuals can discriminate, compensates for both potential end aversion and loss of data, and reduces floor or ceiling effects [19]. The questionnaire was piloted to a convenience sample of front-line and administrative staff working with the investigators to check for clarity, readability and question relevance as well as time for completion of the questionnaires. Suggestions were incorporated into the final version where appropriate.

### Statistical Analysis

Descriptive statistics were calculated for facility characteristics and distributions of item scores. Descriptive data of the item scores were reported as percentages for both the RN and PSW groups. All statistical analyses were conducted on a personal computer running Windows XP Professional using SAS 9.12 statistical software.

## Results

A total of 57 nurses were surveyed from the 8 LTC facilities. This group consisted of 20 registered nurses, 30 registered practical nurses, 1 nurse practitioner, 1 nurse manager, 1 nurse educator, 2 charge nurses, and 2 directors of nursing care. Of the 57 nurses, 48 were responsible for medication administration to the patients, and thus were included in the analysis of bisphosphonate administration. However, all 57 nurses were included in the analysis of the secondary question, assessing their attitudes and beliefs of vitamin D supplementation in LTC. Also surveyed were 124 PSWs employed by the LTC facilities. Of this total, 23 PSWs were responsible for administering medications to LTC patients and were included in the analysis assessing bisphosphonate administration. However, all 124 PSWs were included in the analysis of the secondary question. The average age of the LTC staff surveyed was 45.2 years, with an average of 12.7 years experience working in the LTC setting, and 3.6 years of post-secondary education. The completion rate was 100%, as the project coordinator was present when the surveys were distributed, and waited for all of the RNs and PSWs to complete the survey.

The questionnaire assessed the nursing staff's education to properly administer bisphosphonates by having them select all applicable responses from a list of options (Table 1). These options included administering the drug before meals, after meals, with meals, given with other medications, separated from other medications, given with juice, with or without water, given with the patient sitting up, or while lying down. Only 52% (25 out of 48) of the nurses administered the drug properly, where they selected the options: (given before meals, with water, separated from all other medications, and in an upright position) (Table 1). If at least one incorrect option was selected, it was considered inappropriately administered. For nurses who administered bisphosphonates inappropriately, the most common mistake was administering the bisphosphonates concurrently with other medications, which was seen in 10.4% (5 out of 48) of the nurses surveyed. In addition, 4.2% (2 out of 48) of the nurses gave the bisphosphonates after meals, 2.1% (1 out of 48) gave it with meals, and 2.1% (1 out of 48) each gave the medication while the resident was lying down, with juice, or without water. Upon review of the data collected from the PSWs, only 8.7% (2 out of 23) of those surveyed administered the bisphosphonate correctly. Like the nurses, the most common mistake was administering the bisphosphonate concurrently with other medications, seen in 26.1% (6 out of 23) of PSWs. In addition, 17.4% (4 out of 23) each administered the drug with meals and after meals, 4.4% (1 out of 23) administered it without water, and 21.7% (5 out of 23) administered it with juice. Finally 13% of the PSWs administered the drug while the patient was lying down.

**Table 1:** The assessment of bisphosphonate administration techniques in nurses and personal support workers in the long-term care setting.

Assessment of the proper administration of bisphosphonates		
	**RN Response Rate (n = 48)**	**PSW Response Rate (n = 23)**

Appropriate use of bisphosphonates	52.1% (25/48)	8.7% (2/23)

Given before meals	85.4% (41/48)	30.4% (7/23)

Given after meals	4.2% (2/48)	17.4% (4/23)

Given with meals	2.1% (1/48)	17.4% (4/23)

Given with other medications	10.4% (5/48)	26.1% (6/23)

Given separate from other medications	70.8% (34/48)	17.4% (4/23)

Given with juice	2.1% (1/48)	21.7% (5/23)

Given with water	89.6% (43/48)	39.1% (9/23)

Given without water	2.1% (1/48)	4.4% (1/23)

Given while the resident is sitting up	79.2% (38/48)	39.1% (9/23)

Given while the resident is lying down	2.1% (1/48)	13.0% (3/23)

Do not know (Did not select any available options)	2.1% (1/48)	43.5% (10/23)

The secondary question of this study was to assess the nurse's and PSW's attitudes and knowledge regarding the benefits provided by Vitamin D supplementation (Table 2). The survey included 5 questions, 1 of which assessed the nurse's attitudes regarding if their patients should receive Vitamin D supplementation, and the other 4 assessed their knowledge regarding Vitamin D physiology and benefits. The questions were scored on a 7 point scale (with 1 point corresponding to strongly disagree with the statement, and 7 points corresponding to strongly agree). Of the 57 nurses surveyed, 68% (39 out of 57) strongly felt their patients should be prescribed vitamin D supplements. Our definition of "strongly agrees" corresponded to a score of either 6 or 7 on the 7-point scale. Regarding the nurse's knowledge on Vitamin D, 96.5% (55 out of 57) believed vitamin D improves calcium absorption, 40.3% (23 out of 57) believed it reduces falls, 42.1% (24 out of 57) believed it improved balance, and 68.4% (39 out of 57) believed it reduced fracture rates. The PSW population was also surveyed with the same questionnaire. Of the 124 PSWs who completed the survey, 44.4% strongly felt their patients should be prescribed vitamin D supplementation. From that total, 86.3% believed vitamin D improves calcium absorption, 29.8% believed it reduces falls, 39.5% believed it improved balance, and 56.5% believed it reduced fracture rates.

**Table 2:** Beliefs of nurses and personal support workers regarding vitamin D physiology and benefits.

Beliefs regarding benefits of vitamin D supplementation		
	**RN Response Rate (n = 57)**	**PSW Response Rate (n = 124)**

**Help with absorption of calcium**	96.5% (55/57)	86.3% (107/124)

**Reduce falls**	40.4% (23/57)	29.8% (37/124)

**Help with balance**	42.1% (24/57)	39.5% (49/124)

**Reduce fractures**	68.4% (39/57)	56.5% (70/12)

## Discussion

Osteoporosis management in the LTC facility is not optimal. This is an important issue since LTC residents have a higher incidence of osteoporosis and a higher rate of fracture. Aside from the great impact on the patient's health-related quality of life, fractures also pose a great economic consequence, which poses a large burden on the healthcare system. The estimated one year cost to the healthcare system for a hip fracture sustained in a LTC facility is about $33,000 (Canadian) [20].

Our study shows that at least for the LTC facilities in the Hamilton area, there is still much work to be done in regards to educating the front-line workers. The proper administration of bisphosphonates poses important implications in regards to both drug efficacy, and in preventing rate of adverse events. Only 52% of nurses surveyed administered the bisphosphonates properly. However, this is significantly better than the personal support workers who also administer medications, who had an alarming rate of 8.7% who correctly administer the medication. Bisphosphonate administration is complex, and it must be taken with a full glass of water, while fasting, and the patient must remain in an upright position for 30 to 60 minutes after administration for prevention of esophageal erosion and ensuring adequate absorption. In residents who are bed bound, and unable to remain in an upright position, or have swallowing difficulties, the risk of harm out-weighs the benefits of treatment.

Bisphosphonates have a well-established role in the treatment of patients with osteoporosis. They have been shown to improve both BMD values and also provide a reduction in both vertebral and non-vertebral fracture rates in community dwelling osteoporotic patients [21]. Currently, there is no clear evidence supporting the efficacy of bisphosphonate use in the LTC population, but there has been some data which suggests it may be beneficial. Greenspan et al assessed the use of a bisphosphonate in the LTC population [22]. This trial randomized 327 patients from 25 LTC facilities to either treatment with alendronate 10 mg daily, or placebo. The alendronate group had a significantly greater increase in BMD at 24 months follow up compared to the placebo group at both the lumbar spine (4.4% difference [95% CI 3.3-5.5%]) and femoral neck (3.4%, [95% CI 2.3-4.4%]) [20]. However, this study was not powered to detect a difference in fracture rates. Furthermore, in a recent Cochrane systematic review and meta-analysis, alendronate was shown to reduce the risk of hip fracture in high risk secondary prevention populations [23]. From this, it can be extrapolated that bisphosphonates may reduce hip fractures when used in the LTC population for secondary prevention [24]. There is currently no evidence to show what impact improperly administered bisphosphonates have on the drug's efficacy in regards to impact on BMD values or in fracture prevention. However, this will be an interesting and important clinical question, particularly in this specific population.

Our study also found that there is significant work to be done in educating LTC frontline staff on strategies and therapies that can reduced fracture rates in their patients. We found that only 68% of nurses and 44.4% of PSWs surveyed strongly felt their patients should be prescribed vitamin D supplements. Similarly, based on the surveyed responses, their knowledge regarding vitamin D's benefits and physiology could also be improved through educational initiatives.

Improving the front-line staff's knowledge regarding fracture prevention, falls prevention, and benefits of known therapies may change clinical practice, is also an area for future focus. As an example, our group conducted a study assessing the use of educational protocols to improve the knowledge of family physicians in regards to evidence-based osteoporosis management and fracture risk factors [25]. After 1 year, the family physician's awareness of their patient's risk factors increased, and the utilization of bone mineral density testing in the high risk fracture group significantly increased as well. Similarly, educational programs can be established for nursing staff and other front line LTC members to increase knowledge about fracture risk, prevention, and proper management LTC patients, and this is an area of future research interest for our group.

There are multiple factors attributing to this higher fracture rate, and it is not solely accounted for by the higher incidence of osteoporosis in this population. The LTC population suffers from a higher rate of falls, which is a risk factor for incurring fractures [26,27]. About 1 in 3 individuals above the age of 75 will incur a fall each year [28]. This population also has a higher rate of dementia, which is associated with an increased risk of falls [29], and also has a higher prevalence of vitamin D deficiency [30]. Furthermore, compared to individuals living in the community, those living in LTC facilities are less likely to recover functional capacity after suffering a hip fracture [31]. Predictors of osteoporosis management in LTC residents have been reported. Factors negatively associated with LTC residents receiving osteoporosis therapy include having six or more co-morbidities, wheel chair use, depression, cognitive impairment [30]. It is important to identify risk factors for fractures in this population, and barriers to care, but the next step is to address these issues. Many of these risk factors can targeted, so similar education initiatives as described above can be implemented to educate the LTC staff, and may lead to a change clinical practice.

Prior studies have shown there is a definite care gap between current recommendations compared to the actual practices in these LTC facilities. Calcium and Vitamin D supplementation have been shown to have significant benefits in preventing hip fractures and falls in LTC residents [32]. However, even though this is quite well established and accepted, there were an alarmingly low percentage of LTC residents receiving either of these supplementations. A Canadian study assessing 3 LTC facilities found calcium supplementation in 26% of patients, and Vitamin D supplementation in 30% of patients [33]. The rates in the USA are slightly higher, but are still not up to expected rates [34]. Furthermore, a recent retrospective review of 17 Canadian LTC facilities found that among residents over 65 years old with a prior diagnosis of osteoporosis, or documented fracture (either a prior vertebral fracture or any fracture within 180 days), only 38% of these residents were on a bisphosphonate [35]. Also, only 27.3% of these residents were on vitamin D and calcium supplements [35]. Another study reviewing the health records of 67 LTC facilities in North Carolina and Arizona examined residents with a prior diagnosis of osteoporosis or having sustained a recent hip fracture. The results revealed a similar care gap, revealing calcium and vitamin D supplementation in 69% of residents and bisphosphonate use in only 19% of residents [36]. There were wide variations among facilities which ranged from 0-85% [36]. An earlier American study showed rates of only 11.5% of LTC patients receiving a bisphosphonate, but their data collection period spans from 1995-2004 [21], in contrast to the two aforementioned studies which collected data from 2005-2006 and 2003-2004 respectively. This may suggest that although a care gap indeed still exists, it may have improved compared to the situation in the 1990's. Finally, a recent literature review also showed that the majority (96%) of studies on osteoporosis treatment was conducted on community-dwelling patients, and often the exclusion criteria prevented patients who resemble the LTC population from participating in these studies [37]. This implies that the majority of research on osteoporosis treatment has not focused on the LTC population, and although we often still apply these practices to the LTC group, more dedicated research should focus this particularly high risk group.

Some limitations of our study included that the study was not randomized, the relatively small sample size analyzed, and the geographic limitation of survey distribution to only the Hamilton area. Another potential bias is there may be site specific variability regarding education among the 8 LTC facilities surveyed. Although none of the 8 facilities provided specific education to their staff regarding fracture prevention and osteoporosis treatment, there can still be site specific variability regarding non-dedicated education. However, the results of this study still does shed light on our current situation regarding the osteoporosis care gap, and may lead to further research into this particular area in the near future.

Over the last few years, there have been studies emerging to attempt to understand the barriers to implementing the clinical practice guidelines for osteoporosis care in the LTC population, and further research needs to be continued in this area. This is quite encouraging, and only after understanding current practices and existing care gaps will the development of useful and well accepted knowledge transfer and guideline implementation strategies will be possible.

Areas of future research include implementing some educational initiatives in the LTC facilities and following up to assess if there is indeed an improvement in knowledge regarding fracture prevention and osteoporosis treatment. Another important question to address is if there is indeed an improvement in knowledge, does this also translate to a change in management of the LTC patients.

## Conclusion

Bisphosphonates are quite effective in increasing the bone mineral density of LTC patients, and may reduce fracture rates, but they are only effective if properly administered. Only 52% of the nurses and 8.7% of PSWs surveyed were administering the drug properly. In summary, although the education of health providers has improved since the mid-1990's, this area still requires further attention and the subject of future quality assurance research.

## Competing interests

Dr. Adachi has received consulting fees from Amgen, Astra Zeneca, Eli Lilly, Glaxo-Smith Kline, Merck, Novartis, Pfizer, Procter & Gamble, Roche, Sanofi Aventis, Servier.

A. Papaioannou is or has been a consultant or on the speaker's bureau for the following: Aventis Pharma, Eli Lilly Canada Inc., Merck Frost Canada, Novartis Pharmaceticals Canada Inc., Proctor and Gamble Pharmaceuticals.

All other authors have no financial disclosures.

We would also like to acknowledge funding from the Ontario Ministry of Health and Long-Term Care Osteoporosis Strategy for this project.*

## Authors' contributions

ANL: Role in study design, and drafted the initial manuscript. GI: Performed statistical analysis, and critically appraised review of the initial draft. YP: Role in study design, data collection and critically appraised review of the initial draft. LMG: Role in study design, and critically appraised review of the initial draft. MVH: Role in study design, and critically appraised review of the initial draft. JDA: Role in critically appraisal of the initial draft. AP: Role in study design, and critically appraised review of the initial draft. All authors read and approved the final manuscript.

## Pre-publication history

The pre-publication history for this paper can be accessed here:

http://www.biomedcentral.com/1471-2318/10/73/prepub
